# Acute kidney injury and diabetic kidney disease in children with acute complications of diabetes

**DOI:** 10.1007/s00467-022-05735-7

**Published:** 2022-10-13

**Authors:** Jolanta Soltysiak, Izabela Krzysko-Pieczka, Anna Gertig-Kolasa, Ewa Mularz, Bogda Skowrońska, Danuta Ostalska-Nowicka, Jacek Zachwieja

**Affiliations:** 1 Department of Paediatric Nephrology and Hypertension, Poznan University of Medical Sciences, 27/33 Szpitalna St., 60-572 Poznan, Poland; 2 Department of Paediatric Diabetes and Obesity, Poznan University of Medical Sciences, Poznan, Poland

**Keywords:** Children, Diabetic ketoacidosis, Acute kidney injury, Diabetic kidney disease

## Abstract

**Background:**

Diabetic ketoacidosis (DKA) and hyperglycaemia without ketoacidosis are common acute complications of diabetes. Their association with acute kidney injury (AKI) and diabetic kidney disease (DKD) was studied.

**Methods:**

The study group consisted of 197 children with type 1 diabetes with average diabetes duration of 8.08 ± 2.32 years. The medical history of the patients was retrospectively reviewed. The number of children with severe hyperglycaemia, DKA and AKI was assessed. The association with the risk of chronic kidney disease (CKD) was analysed.

**Results:**

AKI was found in 14% of cases hospitalised for DKA and 8% of cases hospitalised for hyperglycaemia. Patients with AKI showed a significantly increased corrected sodium (141.23 ± 5.09 mmol/L, *p* = 0.035). Patients with AKI in DKA showed a significant increase in WBC (20.73 ± 8.71 × 10^3^/µL, *p* = 0.0009). Follow-up analysis after a minimum of 5 years of diabetes revealed that a single episode of DKA was found in 63 patients and a single episode of AKI in 18 patients. Two or more episodes of DKA were found in 18 patients, and nine cases were complicated by AKI. These patients showed a significant increase in urinary albumin excretion (44.20 ± 64.21 mg/24 h), the highest values of eGFR and the worst glycaemic control.

**Conclusions:**

Diabetic children can develop AKI in the course of DKA and hyperglycaemia without ketoacidosis, which is associated with volume depletion and reflected by corrected sodium concentration. AKI in DKA seems to be complicated by stress and inflammation activation. AKI and poor glycaemic control with repeated DKA episodes can magnify the risk of progression to DKD.

**Graphical abstract:**

**Figure:**
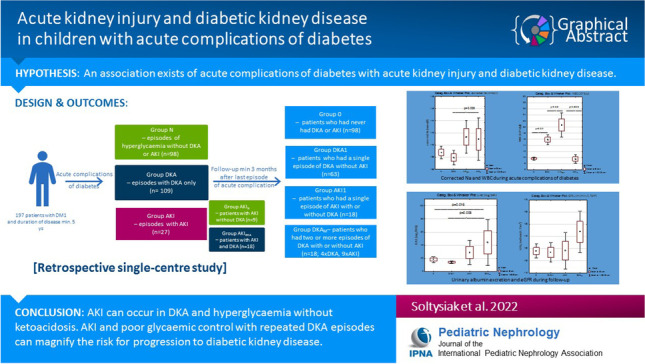
A higher resolution version of the Graphical abstract is available as Supplementary information

**Supplementary Information:**

The online version contains supplementary material available at 10.1007/s00467-022-05735-7.

## Introduction

Diabetic kidney disease (DKD) is a leading cause of chronic kidney disease (CKD), with a high risk of dialysis and mortality [1]. Given the growing incidence of type 1 and type 2 diabetes in children and adolescents, DKD represents a significant public health problem [2, 3]. The pathophysiology of DKD is complex and multifactorial. Chronic and acute hyperglycaemia associated with diabetes leads to glomerular hypertrophy, glomerulosclerosis, tubulointerstitial inflammation and fibrosis. These result in the natural history in DKD of glomerular hyperfiltration, progressive albuminuria, declining glomerular filtration rate (GFR) and ultimately, kidney failure [1]. The earliest alterations in the kidney structure are apparent within 1.5–2 years of type 1 diabetes diagnosis, in the form of a thickening of the glomerular basement membrane. Mesangial volume expansion is detectable within 5–7 years after diabetes diagnosis and then increased albuminuria can also occur [4]. Although DKD is considered a glomerular disease, a growing body of evidence suggests that tubular-interstitial injury may be the first alteration in DKD [5].

The common risk factors for DKD include age, age at onset, duration of diabetes, genetics, gender, glycaemic control, blood pressure, cholesterol levels and smoking [6]. Another risk factor for CKD is acute kidney injury (AKI). Paediatric AKI is associated with increased morbidity and mortality [7–9]. For those who survive AKI, recent data suggest that they likely have permanent kidney damage. These findings have challenged the previous belief that AKI was a completely reversible event [10]. AKI can also occur in diabetes [11]. Acute hyperglycaemic events, chronic poor glycaemic control, and diabetic ketoacidosis (DKA) can lead to AKI [11, 12]. Hyperglycaemia has been shown to induce kidney inflammation and tubulopathy, and poor glycaemic control can lead to polyuria with resultant volume contraction and hypovolemia, which is subsequently associated with the development of pre-renal AKI [13, 14].

DKA is a common and severe acute complication of diabetes. It is characterised by a combination of hyperglycaemia, metabolic acidosis and the production of ketone bodies [15–18]. DKA is currently the leading cause of hospitalisation, morbidity and mortality in youth with type 1 diabetes [15, 19, 20]. Severe hyperglycaemia associated with DKA leads to osmotic diuresis, dehydration and significant pre-renal AKI [17, 18, 21]. In a study by Hursh et al., up to 64% of children with type 1 diabetes hospitalised for DKA developed AKI [15].

AKI is currently defined by the Kidney Disease Improving Global Outcomes (KDIGO) consensus classification based on conventional serum creatinine and urine output (UO) criteria [22]. However, despite the strict standards of AKI and the marked intravascular volume depletion that occurs in DKA, kidney injury in DKA in children has not been systematically studied. Moreover, the impact of DKA on AKI and chronic diabetic kidney injury has not been studied.

The primary objective of this study was to determine the proportion of children with type 1 diabetes hospitalised for the disease that developed severe hyperglycaemia, DKA and AKI. As a secondary objective, we wanted to determine whether developing DKA and AKI was associated with an increased risk for CKD in children with type 1 diabetes for more than 5 years.

## Materials and methods

### Study design and participants

**Fig. 1:**
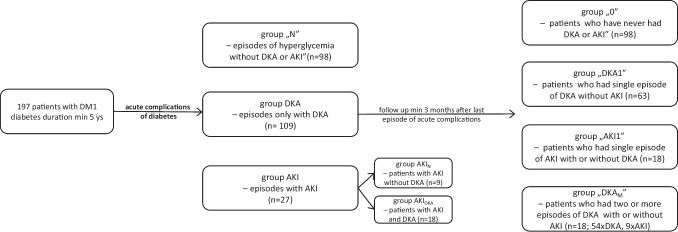
The flow diagram of the study

The study group consisted of 197 adolescents with type 1 diabetes (104 girls and 93 boys) with a mean age of 14.69 ± 2.64 years and with a duration of diabetes of more than 5 years, who were hospitalised in the Department of Paediatric Diabetes and Obesity at Poznan University of Medical Sciences, Poland (Fig. 1). The data was collected in 2019 and 2020. All patients were Caucasian. The medical history of the patients and detailed information, including gender, age, height, weight, body mass index and pre-existing CKD, were retrospectively reviewed based on electronic hospital records. We analysed data obtained at the onset of diabetes and later acute complications, as well as at planned medical control after a minimum of 5 years of diabetes duration. The planned hospitalisation needed to have taken place a minimum of 3 months after the last episode of acute complications of diabetes. However, because in most cases acute complications of diabetes were at the onset of the disease, the observational period was almost as long as the duration of diabetes. No patients had CKD for reasons besides diabetes.

The cases hospitalised due to acute complications of diabetes were divided into the following groups:

- N — cases with hyperglycaemia without DKA or AKI
- DKA — cases with DKA without AKI
- AKI — cases with AKI

On admission, because of acute complications, biological parameters, including pH, HCO_3_, serum creatinine, blood glucose (Glu), serum sodium (Na), haematocrit (HCT) and white blood cell (WBC) count, were collected. The serum creatinine was assessed within 24 h of admission. Corrected sodium (cNa) during DKA episodes was calculated using the following formula:

$$\mathrm{Corrected Na}=\hspace{0.17em}[(\mathrm{Glucose }(\mathrm{mg}/\mathrm{dL})/18)-5.6]\times 0.36+\mathrm{Serum Na}$$

For our patients’ treatment and fluid regimen, we follow the International Society for Pediatric and Adolescent Diabetes (ISPAD) guidelines. Nevertheless, although the resuscitation bolus consisted of 0.9% saline in all our DKA patients, subsequent rehydration in most patients was carried out with balanced salt solution (Optilyte) with the initial maintenance rate of fluid administration as indicated in the guidelines [23].

The biochemical criteria for the diagnosis of DKA were.

- Hyperglycaemia (blood glucose > 11 mmol/L [200 mg/dL])
- Venous pH < 7.3 or serum bicarbonate < 15 mmol/L
- Ketonemia or ketonuria [23]

The severity of DKA was categorised by the degree of acidosis:

- Mild — venous pH < 7.3 or serum bicarbonate < 15 mmol/L
- Moderate — pH < 7.2 or serum bicarbonate < 10 mmol/L
- Severe — pH < 7.1 or serum bicarbonate < 5 mmol/L [23]

AKI was defined according to the KDIGO Clinical Practice Guideline by any of the following:

- Increase in serum creatinine by ≥ 0.3 mg/dL within 48 h
- Increase in serum creatinine to ≥ 1.5 times baseline, known or presumed to have occurred within the prior 7 days
- Urine volume < 0.5 mL/kg/h for 6 h [24]

In acute complications of diabetes, the full age spectrum (FAS) equation (eGFR_FAS_) was used for estimating the glomerular filtration rate [25]. eGFR_FAS_ was calculated using the following formula:

$${\mathrm{eGFR}}_{\mathrm{FAS}}=107.3/(\mathrm{SCr}/\mathrm{Q})$$

SCr: serum creatinine
Q: values [= median serum creatinine in mg/dL] for the FAS equation, according to height-specific healthy populations

Because no study participants had available baseline serum creatinine values before admission at the onset of diabetes or acute complications, we used an estimated GFR of 120 mL/min/1.73 m^2^ to calculate an expected baseline creatinine level (EBC). A GFR of 120 mL/min/1.73 m^2^ was selected based on previously established standards in paediatric AKI studies [26, 27]. Stage 1 AKI occurred if a creatinine value was 1.5 times to less than two times the EBC, stage 2 AKI occurred if a creatinine value was two to less than three times the EBC, and stage 3 AKI occurred if a creatinine value was three times the EBC. KDIGO AKI UO criteria were not used because the recording of hourly UO rates was inconsistent among cases.

During planned medical control after a minimum of 5 years of diabetes duration, the patients were divided into the following groups:

- 0 — patients who had never had DKA or AKI
- DKA1 — patients who had a single episode of DKA without AKI
- AKI1 — patients who had a single episode of AKI with or without DKA
- DKA_M_ — patients who had multiple (two or more) episodes of DKA, including episodes of DKA complicated with AKI

The urinary albumin excretion (UAE), cystatin C, glycosylated haemoglobin (HbA1c) total cholesterol, triglycerides and serum uric acid (UA) were collected. UAE was assessed by 24-h urine collection; kidney function was estimated by glomerular filtration rate (eGFR) according to the Filler formula based on cystatin C [28, 29]; long-term glycaemic control was based on haemoglobin A1c (HbA1c) levels [30]; serum glucose, serum creatinine in blood samples and the examination of albuminuria were measured by an automated biochemical analyser, Alinity c (ABBOTT, USA); WBCs were measured by a blood routine analyser XN-1000 (SYSMEX, Japan); pH was tested in arterial blood by a blood gas analyser ABL 835 (RADIOMETER, Denmark), and HbA1c was measured by glycosylated haemoglobin analyser Alinity c (ABBOTT, USA).

### Statistical analysis

Statistical analysis was performed using Statistica ver. 8 (StatSoft, Tulsa, OK) and MedCalc. The statistical analysis results of the studied parameters were normally distributed and expressed as the mean ± standard deviation (SD). Continuous variables were tested using the analysis of variance Scheffe post hoc tests. The level of statistical significance was *p* < 0.05.

## Results

### Analysis of acute complications of diabetes (Tables 1 and 2; Fig. 2)

**Table 1:** Clinical and laboratory data of the study group during acute complications of diabetes

	*N* (*n* = 98)	DKA (*n* = 109)	AKI (*n* = 27)	p1	p2	p3
Age (ys)	6.56 ± 2.89	8.87 ± 4.66	6.71 ± 4.26	0.0001	NS	NS
Glu (mg/dL)	432.17 ± 171.96	411.19 ± 168.72	460.27 ± 171.13	NS	NS	NS
pH	7.39 ± 0.037	7.22 ± 0.12	7.27 ± 0.11	< 0.000001	< 0.001	NS
HCO_3_ (mmol/L)	20.91 ± 2.54	8.76 ± 3.95	11.92 ± 6.02	< 0.000001	< 0.001	0.00043
HCT (%)	36.92 ± 3.11	40.30 ± 7.48	38.66 ± 3.01	0.00006	NS	NS
cNa (mmol/L)	138.76 ± 4.82	138.95 ± 5.73	141.23 ± 5.09	NS	NS	0.035
WBC (10^3^/µL)	9.65 ± 2.66	15.92 ± 8.32	16.98 ± 8.94	< 0.000001	< 0.0001	NS

*Group N* patients with hyperglycaemia without DKA or AKI, *DKA* patients with DKA without AKI, *AKI* patients with AKI (with or without DKA), *p1* confidence level between DKA and Group N, *p2* confidence level between AKI and Group N, *p3* confidence level between AKI and DKA, *NS* not significant

**Table 2:** Clinical and laboratory data of patients with AKI in DKA and with hyperglycaemia events

	AKI_DKA_ (*n* = 18)	AKI_N_ (*n* = 9)	*p*
Age (ys)	7.56 ± 4.83	4.90 ± 1.86	NS
Glu (mg/dL)	445.68 ± 161.18	499.16 ± 205.35	NS
pH	7.21 ± 0.80	7.38 ± 0.20	< 0.0001
HCO_3_ (mmol/L)	8.22 ± 2.92	19.71 ± 1.62	< 0.0001
HCT (%)	39.88 ± 2.83	38.73 ± 2.58	NS
cNa (mmol/L)	141.34 ± 5.47	140.97 ± 4.34	NS
WBC (10^3^/µL)	20.73 ± 8.71	9.49 ± 2.08	0.0009

*AKI*_*DKA*_ patients with AKI and DKA, *AKI*_*N*_ patients with AKI without DKA, *p* confidence level between AKI_DKA_ and AKI_N_, *NS* not significant

**Fig. 2:**
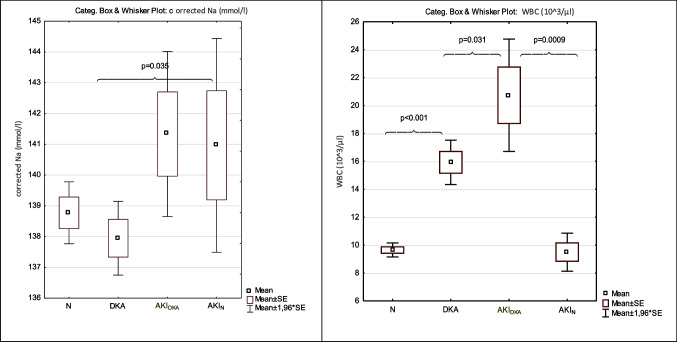
The corrected sodium and WBC levels among groups with acute complications of diabetes. Group N — patients admitted because of hyperglycaemia without DKA; DKA group — patients with DKA; AKI_DKA_ group — patients with AKI during DKA; AKI_N_ — patients with AKI during hyperglycaemia

Retrospective analysis revealed 234 hospitalisations due to acute complications of diabetes. Among them were 98 cases of hyperglycaemia without DKA or AKI.

Diabetic ketoacidosis was diagnosed in 127 cases (54% hospitalisations). In 18 cases, it was complicated with AKI (AKI_DKA_; 14% of cases patients were hospitalised for DKA). In 109 cases of DKA, an episode of AKI occurred.

AKI was diagnosed in 27 cases in total. Apart from 18 episodes of AKI in DKA, nine episodes of AKI occurred in children with hyperglycaemia without DKA (AKI_N_; 8% of cases hospitalised for hyperglycaemic events). The highest prevalence of AKI was noted in patients at the onset of diabetes (22/27 AKI in total). All AKI cases were diagnosed on admission to the hospital. In 21 cases, AKI was in stage 1, and in six cases, it was in stage 2. None were treated in the paediatric intensive care unit (PICU). The presence of AKI did not influence PICU admission. All AKI cases were resolved during the first week of hospitalisation.

Analysis of biochemical parameters during acute complications revealed that patients with AKI showed a significantly increased concentration of corrected sodium (141.23 ± 5.09 mmol/L) when compared to patients who only had DKA. Patients with DKA or AKI showed a significant increase in WBC when compared to group N. The seriousness of acidosis based on pH was comparable in the AKI and DKA groups, whereas the concentration of HCO_3_ was even increased in the AKI group.

Interestingly, the comparison of children with AKI revealed that patients with AKI_DKA_ showed significantly higher WBC levels than children with AKI_N_. Group AKI_N_ presented the highest mean concentration of glucose, but it was not significant (Table 2).

### Analysis of chronic complications of diabetes (Table 3; Fig. 3)

**Table 3:** Clinical and laboratory data of the study group during planned medical control

	Group 0	DKA1	AKI1	DKA_M_	p1	p2	p3	p4
Number of patients	98	63	18	18 (54 × DKA)	na	na	na	na
Number of patients with AKI	0	0	18	8 (9 × AKI)	na	na	na	na
Stage of AKI	na	na	14 × AKI1 4 × AKI2	7 × AKI1 2 × AKI2				
Severity of DKA	na	22 × mild 25 × moderate 16 × severe	8 × normal 2 × mild 6 × moderate 2 × severe	1 × normal (with AKI at onset of the diabetes) 19 × mild 24 × moderate 11 × severe	na	na	na	na
Female/male	53/45	31/32	7/11	12/6 (AKI 5/4)	na	na	na	na
Age (ys) (mean ± SD)	14.22 ± 3.04	13.69 ± 3.16	14.92 ± 3.03	15.62 ± 2.87	NS	NS	NS	NS
Age at onset (ys) (mean ± SD)	6.52 ± 2.87	5.98 ± 3.15	4.31 ± 2.18	7.26 ± 2.79	NS	NS	0.032	NS
Duration of diabetes (ys) (mean ± SD)	7.70 ± 2.22	7.71 ± 2.02	10.60 ± 2.61	8.21 ± 2.18	NS	NS	0.018	< 0.0001
BMI (kg/m^2^) (mean ± SD)	20.23 ± 3.61	20.38 ± 3.25	20.57 ± 3.82	22.94 ± 4.16	0.035	NS	NS	NS
UAE (mg/24 h) (mean ± SD)	18.25 ± 21.78	14.16 ± 7.89	29.03 ± 36.94	44.20 ± 64.21	0.016	0.005	NS	NS
eGFR_F_ (mL/min/1.73 m^2^) (mean ± SD)	127.97 ± 28.57	126.90 ± 32.49	128.34 ± 27.73	143.79 ± 32.31	NS	NS	NS	NS
HBA1c (%) (mean ± SD)	7.29 ± 1.09	7.58 ± 1.44	7.50 ± 1.27	10.29 ± 3.86	< 0.0001	< 0.0001	0.0003	NS
Chol (mg/dL) (mean ± SD)	179.95 ± 32.89	185.41 ± 30.17	191.18 ± 35.00	220.39 ± 71.04	0.0006	0.007	NS	NS
TG (mg/dL) (mean ± SD)	73.05 ± 31.79	81.46 ± 39.41	98.23 ± 66.97	225.44 ± 155.04	< 0.0001	< 0.0001	< 0.0001	NS
UA (mg/dL) (mean ± SD)	4.05 ± 1.04	4.17 ± 0,95	4.44 ± 1.27	4.52 ± 1.14	NS	NS	NS	NS

*NS* not significant, *na* not applicable, *Group 0* patients who had never had DKA, *DKA1* patients with a single episode of DKA during diabetes duration without AKI, *AKI1* patients with a single episode of DKA complicated by AKI, *DKA*_*M*_ patients with multiple (two or more) episodes of DKA, *p1*confidence level between DKA_M_ and Group 0, *p2* confidence level between DKA_M_ and DKA1 subgroup, *p3* confidence level between DKA_M_ and AKI1 subgroup, *p4* confidence level between AKI1 and DKA1 subgroup

**Fig. 3:**
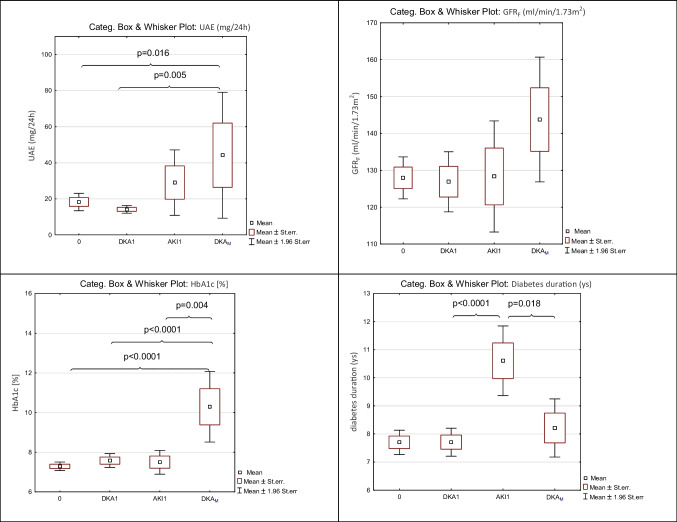
UAE, GFR_F_, HbA1c and diabetes duration among groups in children after 5 years’ duration of diabetes. Group 0 — patients who had never had DKA; DKA1 — patients with a single episode of DKA during diabetes duration without AKI; AKI1 — patients with a single episode of DKA complicated by AKI; DKA_M_ — patients who had multiple (two or more) episodes of DKA during diabetes duration

Among the 197 patients with diabetes, 91 had DKA (46%). A single episode of DKA was diagnosed in 73 patients (37%), usually at the onset of the disease. In 63 patients, it was a single episode of DKA without AKI (group DKA1).

In 18 cases (group AKI1), a single episode of AKI was diagnosed (in 10 cases during DKA and in eight cases during severe hyperglycaemia).

Two or more episodes of DKA were found in 18 patients (9%, group DKA_M_). In group DKA_M_ there were 54 episodes of DKA in total, including nine episodes of AKI in eight patients. A girl who had seven episodes of DKA showed two episodes of AKI.

In 98 patients (49%), no episodes of DKA or AKI occurred (group 0).

The UAE was significantly increased in group DKA_M_ (44.20 ± 64.21 mg/24 h) compared to groups 0, DKA1, and AKI1. The AKI1 subgroup showed an increase in UAE levels, but it was not significant.

The eGFR did not differ much among the studied groups. However, the highest values were in DKA_M_. Group DKA_M_ had the worst glycaemic control and the highest levels of lipids (total cholesterol and triglycerides). The duration of diabetes was significantly increased only in AKI1, and these patients were the youngest group at the onset of diabetes.

## Discussion

This study revealed that among all acute complications of diabetes, around half of the patients had DKA, mostly a single episode. It usually occurred at the onset of the disease. This is consistent with other reports, which show frequencies of DKA at the beginning of diabetes from approximately 15 to 70%, depending on the age at diagnosis, origin, ethnicity, and access to medical care. The risk of DKA in established type 1 diabetes is estimated to be between 1 and 10% in each patient every year, similar to the findings of our study, in which 9% of established patients showed two or more episodes of DKA [31, 32].

However, our study revealed that only 14% of children hospitalised for DKA developed AKI. This is much less than what other authors have reported. Hursh et al. presented that 64.2% of children hospitalised for DKA had AKI, mostly stage 2 [15]. Baalaaji et al. showed AKI in 35.4% of children with DKA admitted to a single PICU. In contrast, Myers et al. showed that 43.0% of children with DKA had AKI (1359 episodes) [33, 34]. In a recently published study by Al Khalifah et al., the AKI incidence reached 80.75% of all children with DKA. However, none of these studies investigated the development of AKI during hyperglycaemia without ketoacidosis.

Baalaaji used the pRIFLE classification based on decreased estimated creatinine clearance [27]. Using the Schwartz formula, Hursh, Myers and Al Khalifah used serum creatinine measurements using the KDIGO criteria and eGFR of 120 mL/min/1.73 m^2^ to calculate an expected baseline creatinine level. In this study, we used the FAS formula as it has more validity for patients with different heights or ages. Nevertheless, the percentage of AKI in DKA was still low compared to other studies.

In the present study, all patients with AKI showed a significantly increased corrected sodium, considered a good indicator of dehydration in diabetes. In many studies, the corrected sodium was increased in AKI patients and usually correlated with the severity of AKI. All studies analysed AKI in diabetic ketoacidosis [15, 16, 21, 33, 34].

Interestingly, our study revealed that AKI can also occur in children without DKA during severe hyperglycaemia, and it represents 8% of cases of hyperglycaemic events. All AKI_N_ episodes were established at the onset of diabetes and were in stage 1. These children presented the highest mean levels of glucose. In the setting of diabetes, as shown by others, the extracellular volume depletion and pre-renal AKI are commonly induced by glucosuria and osmotic diuresis because of poorly controlled diabetes [15, 35]. Severe hyperglycaemia seems to be enough to cause AKI.

In our study, children with AKI during DKA above increased corrected sodium also showed the highest WBC concentration (20.73 ± 8.71 × 10^3^/µL). An increased WBC was noticed in an earlier study in diabetic adults, in which AKI patients showed a WBC of 16.51 × 10^3^/µL vs. 9.38 × 10^3^/µL in DKA without AKI [36].

Leucocytosis is very common in hyperglycaemic crises, but its origin is still unknown [23]. It seems to respond to metabolic stress during DKA without apparent infection [37]. Significant dehydration, haemoconcentration and hyperglycaemia lead to the release of catecholamine and cortisol from adrenal glands, increasing leucocyte levels [38–40]. Moreover, a lack of insulin and a lack of its possible anti-inflammatory effect can stimulate the production of neutrophils in bone marrow [41].

In addition to increased WBC, the elevation of cytokines such as TNF-α and IL-6 can also occur in DKA [42]. These cytokines, along with IL-1β, regulate the production of acute-phase proteins [43]. In DKA, increased reactive oxygen species production leads to increased cytokine levels and the emergence of growth factor receptors [44]. Cytokines released during DKA may result in capillary perturbation and thus may contribute to developing acute clinical complications (i.e., cerebral or pulmonary oedema). The pathophysiology of these complications remains uncertain, but they likely involve some capillary perturbation that begins before the management of DKA and is accentuated by it [45].

In other words, increased WBC may reflect the severity of stress and inflammatory activation during DKA, resulting in capillary perturbation and AKI. The combination of poor glycaemic control with pre-renal AKI and inflammatory activation during DKA can intensify AKI in DKA. In our study, the most severe AKI (stage 2) was diagnosed only in patients with DKA.

AKI is an independent factor associated with more extended hospital stays and a higher mortality rate for children [46]. However, AKI is also associated with long-term health outcomes. In a review that included 13 cohort studies of adults with AKI, a single episode of AKI was associated with an increased risk of developing CKD, with a pooled adjusted hazard ratio of 8.8 (95% CI, 2.1–25.5) [11].

In the present study, children with AKI (groups AKI1 and DKA_M_) showed the highest concentration of albuminuria during planned medical control. Moreover, in group DKA_M_, changes in eGFR were noticed and were highest, reflecting the tendency to hyperfiltration, which is often the initial sign of DKD [4].

The development of DKD is associated with many alterations in the structure of multiple kidney compartments, which can start very early, even within 1.5–2 years of diabetes diagnosis. It is paralleled by capillary and tubular basement membrane thickening. Other glomerular changes include the loss of endothelial fenestrations, mesangial matrix expansion and loss of podocytes with effacement of foot processes. The longer the duration of diabetes, the higher the risk of DKD [4]. In the present study, the mean diabetes duration was 8 years. However, only patients with AKI or more than two episodes of DKA with poor glycaemic control showed changes in UAE. This emphasises that AKI and repeated episodes of DKA with poor glycaemic control are an essential risk for chronic kidney injury. In particular, the AKI1 group had the longest duration of diabetes (10.60 ± 2.61 years). This finding highlights that the duration of diabetes is an important risk factor for CKD.

Interestingly, the DKA_M_ group showed the highest increase in HbA1c and lipids compared to other groups. In other words, children with repeated DKA showed the worst glycaemic control with the highest risk of CKD and progression to DKD. The AKI incidence in this study might be an underestimation because it is possible that in the DKA_M_ group, there were more episodes of AKI than were diagnosed.

A limitation of this study is the relatively small subgroup with AKI and a short follow-up period. It was also a single-centre study, and further cohort studies are needed to clarify the impact of AKI and DKA on DKD in children.

## Conclusions

Acute complications of diabetes mellitus are risk factors for AKI. This can occur in children with DKA and those with hyperglycaemia without ketoacidosis. AKI incidences in diabetes are associated with volume depletion reflected by corrected sodium concentration. In children with DKA, AKI incidences seem to be complicated by stress and inflammation activation, reflected by increased WBC. AKI and repeated DKA with poor glycaemic control can magnify CKD and progression to DKD. Prospective longitudinal studies are needed to better understand the risk factors and long-term implications of AKI and DKA in children with diabetes.

## Supplementary Information

Below is the link to the electronic supplementary material.

Graphical Abstract (PPTX 95 KB)

Supplementary file2 (DOCX 33 KB)
