# Supplementary material for: Acute kidney injury and diabetic kidney disease in children with acute complications of diabetes
Source: Pediatr Nephrol. 2022 Oct 13;38(5):1643–52. doi: 10.1007/s00467-022-05735-7 (PMC10060302; doi:10.1007/s00467-022-05735-7)
Supplement: Supplementary file 1 — Graphical Abstract (PPTX 95 KB) [file 467_2022_5735_MOESM1_ESM.pptx]

## Slide 1
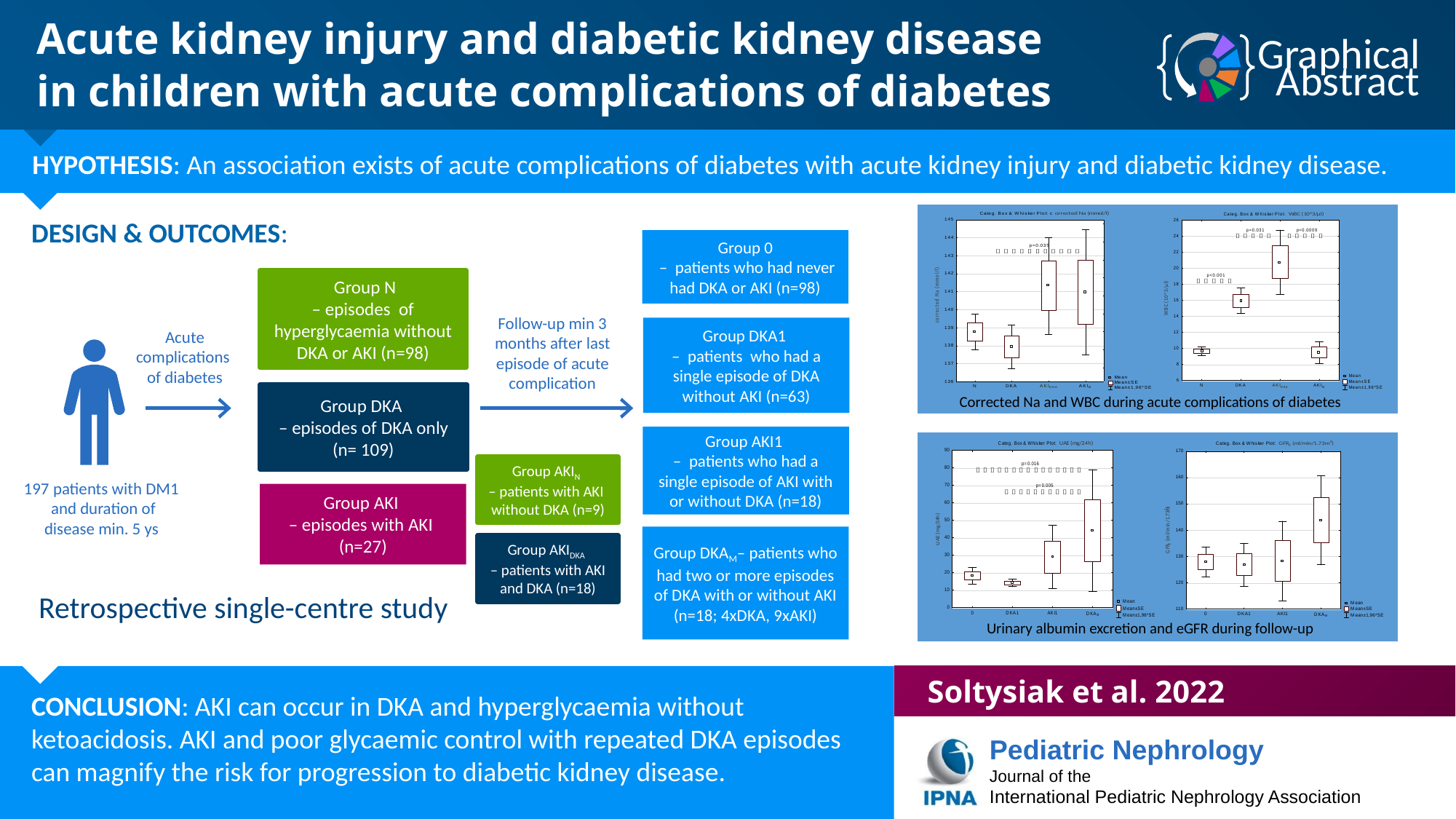

Acute kidney injury and diabetic kidney disease
in children with acute complications of diabetes
HYPOTHESIS: An association exists of acute complications of diabetes with acute kidney injury and diabetic kidney disease.
| |
| --- |
DESIGN & OUTCOMES:
Group 0
 – patients who had never had DKA or AKI (n=98)
 Group N
– episodes of hyperglycaemia without DKA or AKI (n=98)
Follow-up min 3 months after last episode of acute complication
Group DKA1
– patients who had a single episode of DKA without AKI (n=63)
Acute complications
of diabetes
Group DKA
– episodes of DKA only
(n= 109)
Corrected Na and WBC during acute complications of diabetes
Group AKI1
– patients who had a single episode of AKI with or without DKA (n=18)
| |
| --- |
Group AKIN
– patients with AKI
without DKA (n=9)
197 patients with DM1
 and duration of disease min. 5 ys
Group AKI
– episodes with AKI
(n=27)
Group DKAM– patients who had two or more episodes of DKA with or without AKI (n=18; 4xDKA, 9xAKI)
Group AKIDKA
– patients with AKI
and DKA (n=18)
Retrospective single-centre study
Urinary albumin excretion and eGFR during follow-up
Soltysiak et al. 2022
CONCLUSION: AKI can occur in DKA and hyperglycaemia without ketoacidosis. AKI and poor glycaemic control with repeated DKA episodes can magnify the risk for progression to diabetic kidney disease.
